# The Future Glioblastoma Clinical Trials Landscape: Early Phase 0, Window of Opportunity, and Adaptive Phase I–III Studies

**DOI:** 10.1007/s11912-023-01433-1

**Published:** 2023-07-04

**Authors:** Nicholas S. Cho, Weng Kee Wong, Phioanh L. Nghiemphu, Timothy F. Cloughesy, Benjamin M. Ellingson

**Affiliations:** 1 UCLA Brain Tumor Imaging Laboratory, Center for Computer Vision and Imaging Biomarkers, Department of Radiological Sciences, David Geffen School of Medicine, University of California Los Angeles, 924 Westwood Blvd., Suite 615, Los Angeles, CA 90024 USA; 2 Department of Radiological Sciences, David Geffen School of Medicine, University of California Los Angeles, Los Angeles, CA USA; 3 Department of Bioengineering, Henry Samueli School of Engineering and Applied Science, University of California Los Angeles, Los Angeles, CA USA; 4 Medical Scientist Training Program, David Geffen School of Medicine, University of California Los Angeles, Los Angeles, CA USA; 5 Department of Biostatistics, Fielding School of Public Health, University of California Los Angeles, Los Angeles, CA USA; 6 Department of Neurology, David Geffen School of Medicine, University of California Los Angeles, Los Angeles, CA USA; 7 Department of Neurosurgery, David Geffen School of Medicine, University of California Los Angeles, Los Angeles, CA USA; 8 Department of Psychiatry and Biobehavioral Sciences, David Geffen School of Medicine, University of California Los Angeles, Los Angeles, CA USA

**Keywords:** Glioblastoma, Clinical trials, Phase 0 trials, Window of opportunity trials, Adaptive trials

## Abstract

**Purpose of Review:**

Innovative clinical trial designs for glioblastoma (GBM) are needed to expedite drug discovery. Phase 0, window of opportunity, and adaptive designs have been proposed, but their advanced methodologies and underlying biostatistics are not widely known. This review summarizes phase 0, window of opportunity, and adaptive phase I–III clinical trial designs in GBM tailored to physicians.

**Recent Findings:**

Phase 0, window of opportunity, and adaptive trials are now being implemented for GBM. These trials can remove ineffective therapies earlier during drug development and improve trial efficiency. There are two ongoing adaptive platform trials: GBM Adaptive Global Innovative Learning Environment (GBM AGILE) and the INdividualized Screening trial of Innovative GBM Therapy (INSIGhT).

**Summary:**

The future clinical trials landscape in GBM will increasingly involve phase 0, window of opportunity, and adaptive phase I–III studies. Continued collaboration between physicians and biostatisticians will be critical for implementing these trial designs.

## Introduction

Glioblastoma (GBM) is the most common primary malignant brain tumor and remains incurable. Prognosis is poor as median progression-free survival remains around 6.9 months and median overall survival remains around 14.6 months [1]. Despite advancements in characterizing GBM pathogenesis and potential therapeutic vulnerabilities, the standard of care for newly diagnosed GBM of maximally safe surgery followed by radiation therapy with concurrent and adjuvant temozolomide chemotherapy has remained largely unchanged for decades [2]. Upon recurrence, only about 1 in 4 patients can undergo repeat surgery due to concerns of morbidity [3], and other treatment options include repeat chemoradiation, anti-angiogenic agents (bevacizumab), tumor treating fields therapy, and inclusion into clinical trials.

There have been very few therapies for GBM approved by the United States Food and Drug Administration (FDA) over the past two decades (see review by Fisher et al. [4]) because the clinical translation of novel findings on GBM pathogenesis into drug discovery poses significant challenges. The brain is considered an “immunologically-privileged site” because of the blood-brain barrier (BBB) [5], and the GBM tumor microenvironment causes further immunosuppression [6]. External, direct delivery of therapeutics to the brain is also challenging given the invasive nature of neurosurgical procedures. Difficulty accessing the brain is also a reason why non-invasive imaging, particularly magnetic resonance imaging, is critical for GBM patient management and assessing therapeutic efficacy in GBM clinical trials such as through the Modified Response Assessment in Neuro-Oncology criteria [7].

Recently, concerns have also been raised whether the lack of GBM drug discovery can also stem from the GBM clinical trial landscape needing improvement [8–10]. In fact, development times for GBM clinical trials from just the beginning of phase II studies until the end of phase III studies are on average 7.2 years [9], and 91% of phase III trials fail in GBM [11]. As a result, there have been two recent innovations in GBM clinical trial designs to improve the efficiency of drug discovery timelines: 1) early phase 0 and window of opportunity clinical trials for rapid identification of ineffective therapies and 2) adaptive designs in clinical trials for efficient, and ethical, study designs.

## Phase 0 and Window of Opportunity Clinical Trials in Glioblastoma

### Overview of Phase 0 Clinical Trials

To improve the transition of preclinical drug discoveries into patient care, the US FDA released a guidance in January 2006 on Exploratory Investigational New Drug (xIND) applications that would allow for preliminary assessment on biological efficacy of study drugs before or in parallel to the conventional assessment of drug safety and toxicity in traditional IND applications phase I studies. Kummar et al. proposed the creation of human phase 0 trials for oncology to be conducted under xIND applications [12]. Under traditional IND applications, the first-in-human studies are phase I studies, which are focused on drug safety and toxicity of the study drug in human patients. However, under xIND applications, phase 0 studies assess the biological efficacy of the study drug in human patients using a *non-therapeutic*, but still pharmacologically active dose [12] (Table 1). Phase 0 studies are particularly important for drug development of targeted therapies, where the therapeutic effect relies on successfully reaching the tissue target and causing a pharmacodynamic response. As a result, ineffective therapies that cannot modulate the target tissue can be rapidly removed from further testing through phase 0 studies. Other differences between phase 0 and phase I studies (Table 1) are that phase 0 studies have fewer patients (typically ~10 patients) and require much less pre-clinical data for toxicology results in xIND applications than in traditional IND applications for phase I studies [12, 13].

**Table 1:** Comparison of typical phase I, window of opportunity, and phase 0 clinical trials

Characteristic	Phase I trials	Window of opportunity trials	Phase 0 trials
Primary Endpoint	Determine the MTD	Demonstrate target tissue modulation	Demonstrate target tissue modulation
Dose Level	Therapeutic	Therapeutic but only for brief period (e.g. 1 cycle)	Non-therapeutic (But in neuro-oncology can be therapeutic but only for brief period (e.g. 1 cycle))
Tumor Biopsy	Not required	Required	Required
PK & PD Analyses	Not always performed (sometimes done as Ib study)	Performed	Performed
Sample Size	>15	<15	<15

*MTD* maximum tolerated dose; *PK* pharmacokinetic; *PD* pharmacodynamic

### Phase 0 vs. Window of Opportunity Trials

Of note, the term “phase 0” trial is sometimes interchanged with the term “window of opportunity” trial in neuro-oncology. Both trial designs involve administering a study drug for a short period between the time “window” of the study subject’s date of diagnosis/recurrence and the date of surgery, and then obtaining surgical tissue for assessing pharmacodynamic effects. However, we would like to point out these two concepts are distinctly different and should not be used interchangeably. As described by Aroldi & Lord [14•], traditional phase 0 trials involve *non-therapeutic* microdoses while window of opportunity trials involve therapeutic doses but only for a short duration (e.g., 1 cycle) (Table 1). The briefly administered therapeutic doses in window of opportunity trials are to ensure sufficient drug penetrance so that successful pharmacodynamic assessment can occur. Furthermore, window of opportunity trials are sometimes performed as part of a separate “surgical arm” in later phase I or phase II studies. In the field of neuro-oncology, however, phase 0 studies can be performed in the style of window of opportunity trials in terms of using a higher dose level to ensure BBB penetrance, but use of the maximum tolerated dose is often used for a short period of time to limit potential toxicities [15•]. Hence, phase 0 and window of opportunity studies are sometimes used interchangeably in neuro-oncology, as the designs of such trials can be very similar.

### Specific Considerations for Glioblastoma Phase 0 and Window of Opportunity Clinical Trials

**Fig. 1:**
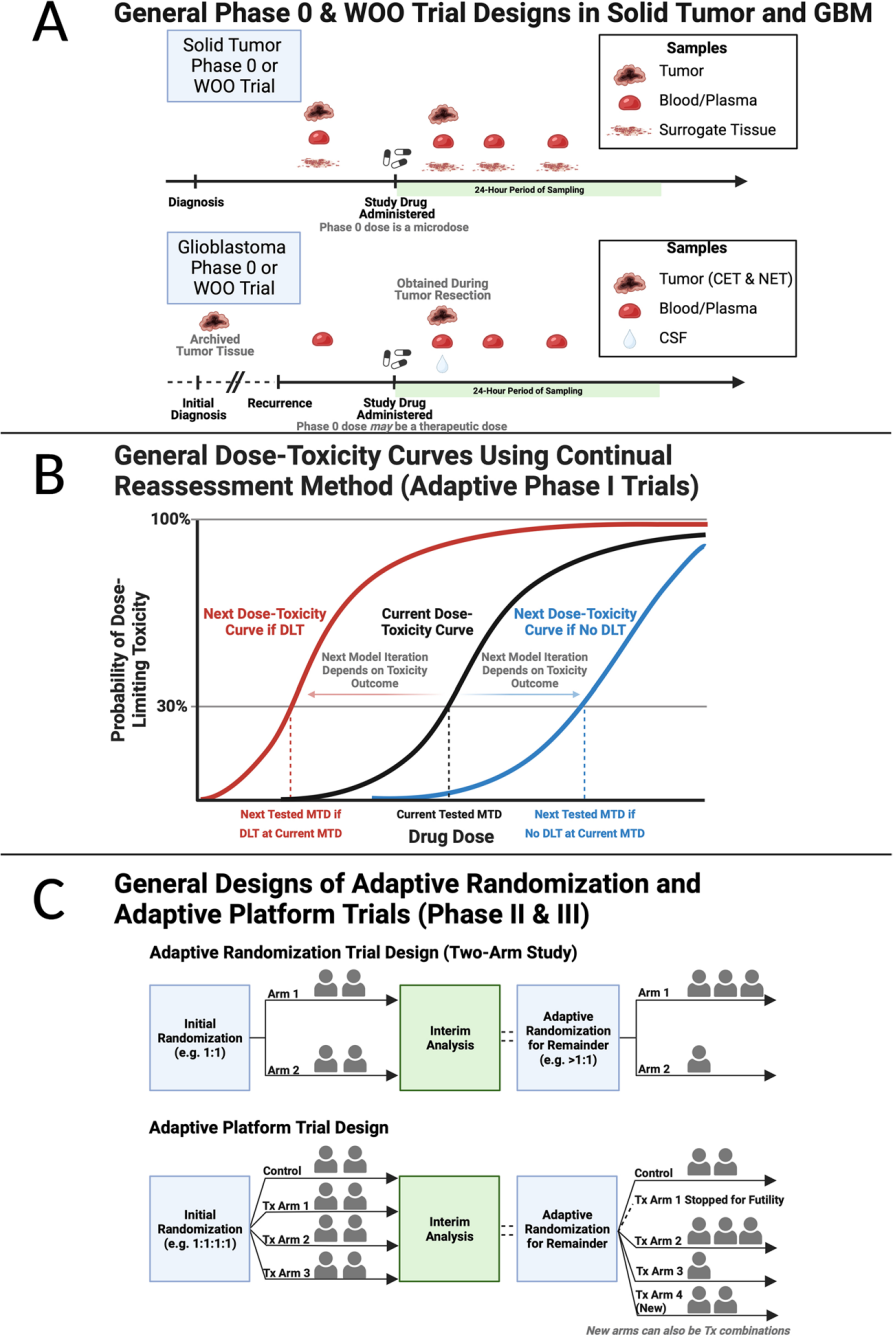
Overview of phase 0, window of opportunity, and adaptive phase I-III study designs in GBM. (**A**) General schematic of differences in phase 0 & window of opportunity studies in solid tumors compared to those in GBM. (**B**) General schematic of dose-toxicity curves using continual reassessment method to estimate the next tested MTD in adaptive phase I trials. (**C**) General designs of adaptive randomization and adaptive platform trials in phase II & III trials. Created with BioRender.com. Adapted from “Mouse Experimental Timeline,” by BioRender.com (2023). Retrieved from https://app.biorender.com/biorender-templates. GBM = glioblastoma; WOO = window of opportunity; CET = contrast-enhancing tumor; NET = non-enhancing tumor; CSF = cerebrospinal fluid; DLT = dose-limiting toxicity; MTD = maximum tolerated dose; Tx = treatment

The general design of phase 0 and window of opportunity studies is shown in Fig. 1A. Before the study drug is administered, tumor biopsies and surrogate tissue biopsies are obtained as a baseline. Then, the study drug is administered at a *non-therapeutic* dose, and post-treatment blood/plasma samples are obtained for pharmacokinetic analyses. Additionally, a post-treatment tumor biopsy and/or surrogate tissue biopsies are obtained for pharmacodynamic analyses, usually with outpatient needle biopsy procedures for non-CNS tumors.

Importantly, there are study design considerations unique to studies on patients with GBM (Fig. 1A). For instance, cerebrospinal fluid (CSF) samples can also be obtained as surrogate tissue to extrapolate BBB penetrance. Also, biopsy samples of GBM can only be acquired as part of an inpatient neurosurgical procedure (usually during craniotomies), so pre-treatment baseline tissue biopsies are unable to be acquired just prior to study drug administration. Instead, archived tumor tissue from prior resections are used as a baseline, but this tissue may be from months to even years in the past [16]. An inherent limitation is that the archived tumor tissue may not represent the tissue that is receiving the study drug since recurrent GBMs are known to have altered tumor microenvironments and genetics compared to newly-diagnosed GBMs [17, 18]. Alternatively, there could be a control arm in matched untreated tissue samples for cross-sectional comparison. During the craniotomy, tumor samples should be obtained from the BBB-permeable contrast-enhancing tumor, which is the most active tumor region in gliomas [19–21], as well as the relatively more diffuse and BBB-intact non-enhancing tumor to capture heterogeneity in intra-tumor microenvironments and BBB penetrance [22]. Lastly, given the invasive nature and long procedural times of craniotomies, there must be careful consideration of the post-treatment surgical timing as well as strong coordination of the entire study team—which includes all operating room personnel—to successfully achieve time-sensitive tissue collection [16].

### Ethical Considerations of Glioblastoma Phase 0 and Window of Opportunity Clinical Trials

There are significant ethical considerations of phase 0 trials since participants may be subjected to risks without any possible therapeutic benefit. These ethical concerns are even further amplified in GBM phase 0 and window of opportunity studies given the highly invasive nature of craniotomies. As a result, for GBM phase 0 and window of opportunity studies, the participant’s disease course must warrant surgery regardless of their participation in the study because the risks of craniotomy are too high for participants for whom a surgery is not warranted [22]. However, the timing of surgery may be delayed for trial enrollment and pre-surgical study drug administration, so patients eligible for these studies must be clinically stable enough so that the delay in surgery does not have significant impact on their management [16]. Participants in a phase 0 study should also remain eligible to participate in other clinical trials that offer the possibility of therapeutic benefit [23].

### Statistical Considerations of Glioblastoma Phase 0 Clinical Trials

Despite numerous advanced statistical methods developed for phase I–III studies, the statistical literature on phase 0 studies remains sparse. Phase 0 studies can be viewed as miniature phase II studies that involve less patients and are assessing for a pharmacodynamic response instead of a clinical response [24]. However, phase 0 studies have limited sample size and there is difficulty in estimating the criteria that would constitute a pharmacodynamic response [24]. Kummar et al. offered preliminary statistical guidelines and example designs for dichotomized pharmacodynamic endpoints (yes/no biological effect) when first introducing phase 0 studies [12]. Murgo et al. extended these designs by providing calculations for determining criteria of pharmacodynamic response and analysis methods [13]. Lastly, Rubinstein et al. provided study design estimates for different pharmacodynamic response rates as well as Simon Optimal and Minimax designs [24, 25].

In practice, however, phase 0 studies on patients with GBM are often deemed as exploratory analyses, so there is often a lack of a priori power analysis to determine sample sizes [15•, 22, 26–28], and low sample sizes are justified based on feasibility [15•, 28]. Response criteria may be determined based on prior pre-/clinical data [26] or simply assessing if there is a relative change compared to archived tissue without a threshold (one-fold difference) [27]. Nevertheless, the relatively recent development of phase 0 studies in GBM may be ripe for developing and implementing novel statistical innovations.

## Adaptive Designs in Glioblastoma Phase I–III Clinical Trials

### Overview of Adaptive Designs

One statistical innovation beginning to be implemented in GBM phase I–III studies is adaptive designs (see reviews on adaptive designs by Rosenberger et al. [29], Dragalin [30], and Sverdlov et al. [31•]). The US FDA also recently released a final guidance on adaptive designs in November 2019 [32], which further underscores the growing interest in this topic. The main motivation for adaptive designs is to improve the efficiency of clinical trials by minimizing patient exposures to ineffective or toxic therapies.

Broadly, adaptive designs in clinical trials involve *prospectively defined* trial modifications based on interim analyses of the trial’s accumulated data; these modifications allow for changes in the study design while maintaining the same statistical rigor and objectives of the initial trial design [30]. The remainder of this review will focus on the application of 1) adaptive dose-finding strategies in GBM phase I studies as well as 2) adaptive stopping rules and 3) adaptive allocation rules (also called *adaptive randomization*) in GBM phase II & III studies.

### Adaptive Dose-Finding Strategies in Glioblastoma Phase I Clinical Trials

Phase I studies are focused on the safety of the study drug, and their main objective is to determine the maximum tolerated dose (MTD) by escalating dose levels until the pre-specified dose-limiting toxicity (DLT) rate is reached (see review by Le Tourneau et al. [33]). Accurate determination of the MTD is critical because the MTD determines the recommended phase II dose (RP2D) for continued trials of the study drug (in the USA, the MTD is the RP2D).

The traditional phase I study design is the 3+3 design [34], and its widespread use is due to its simplicity. However, there are several limitations to the 3+3 design. First, the dose escalation is often slow, so excessive patients may be treated below therapeutic levels [35], and consequently, trial durations may be unnecessarily long. Second, it is difficult to anticipate the final sample size a priori because the study design results in a random sample size. Given this limitation, there is a that a 3+3 design study results in an early termination at a dose level that is not reflective of the true MTD [36, 37].

Given the limitations of 3+3 designs, other algorithm-based approaches have been designed for phase I studies. One example is the modified toxicity probability interval (mTPI) [38]. Here, an equivalence interval (EI) for the MTD is chosen based on the accepted tolerance of DLT rate (e.g., 0.25–0.35 for DLT rate of 0.30). Then, the probability of DLT (0–1) is split into three intervals: 0 to the lower bound of the EI (dose is below the MTD), the EI (dose is close to the MTD), and upper bound of the EI to 1 (dose is above the MTD) [38]. After a cohort is treated at a dose level and their DLT information is recorded, the unit probability mass (UPM) is computed for each of the three intervals. Then, the next dose level is based on the UPM results: escalate if the UPM of underdosed interval is highest; de-escalate if the UPM of overdosed interval is highest; stay at the same level if the UPM of EI is highest. A limitation of the mTPI approach though is that excessive patients may be treated at a toxic dose [37].

Another approach is the Bayesian optimal interval (BOIN) design, which improves on the mTPI design by using advanced statistical methods to calculate a corresponding target DLT *range* for a target DLT (e.g., the BOIN design interval for a DLT target of 0.3 corresponds to 0.236–0.358) [37]. A target maximum sample size is chosen, and the DLT rate is assessed after each cohort of patients and compared to the BOIN range to determine if the dose should be escalated, de-escalated, or maintained for the next cohort. This cycle is repeated until the maximum sample size is obtained. Some advantages of the BOIN design include that patient cohorts do not need to be fixed at groups of 3 and that the traditional 3+3 design is nested within the general BOIN design. However, a limitation of both the BOIN and mTPI designs is that they do not utilize all the previously acquired data to estimate the MTD.

Adaptive dose-finding strategies based on the continual reassessment method (CRM) have been developed for phase I studies that utilize all the prior data to estimate the MTD [35, 39–41]. In the CRM, a dose-toxicity curve model, DLT rate, and a stopping rule (such as total sample size overall or for a dose level) are chosen. Once a patient is treated at the first dose level and their DLT information is recorded, the dose-toxicity curve model is updated to estimate the new MTD (the dose corresponding to the intersection of the dose-toxicity curve and the chosen DLT rate; Fig. 1B). Then, the next patient is treated at the new MTD, their DLT information is recorded, and the model is updated again to find the new MTD. This cycle is repeated until the stopping rule has been met, such as when a specified number of patients have been treated at the same (or within a threshold of a) dose level [35, 41]. The escalation with overdose control (EWOC) method extends on the CRM to penalize overdosing more than underdosing [42] and can be applied to drug combination trials [43]. Modified CRM designs also include larger cohorts per dose level [44]. Some limitations include that there must be some a priori guess of the MTD and of the dose-toxicity curve mathematical model (e.g., hyperbolic, one-parameter logistic, two-parameter logistic [41]). There have also been concerns for rapid dose-escalations using the CRM, so dose-escalation rules have been described [41] as well as the capability of the clinical team to override the CRM’s suggested dose level for select cases [40].

CRM-based adaptive dose-finding methods have started to become more widespread in contemporary GBM phase I trials [45–48]. Cohorts of 3 patients per dose level, restrictions on the next dose level being 150% of the previous dose level, and stopping rules of being within 10% of the prior dose level for two consecutive iterations have been reported [46, 47]. It appears very evident that GBM phase I studies incorporating adaptive dose-finding methods will become more common place in the future.

### Adaptive Randomization and Stopping Rules in Glioblastoma Phase II & III Clinical Trials

In recent years, there has also been growing interest in adaptive randomization and stopping rules in GBM phase II & III clinical trials because these procedures can address the need for improved clinical trial efficiency and the ethical consideration of minimizing the number of patients exposed to ineffective therapies. In adaptive stopping designs, trials may be stopped early for *superiority*, such as when the treatment arm clearly outperforms the control group, or for *futility*, such as when it becomes clear that the study drug will not perform significantly better than the control arm when the trial is terminated. The assessment can be based on predictive probability modeling or by using Bayesian approaches that estimate the posterior probability of treatment success [49]. Adaptive stopping designs would greatly minimize patient exposures to potentially ineffective treatments and, consequently, clinical trial costs.

In adaptive randomization designs, patient allocations to trial arms are allowed to deviate based on interim results instead of traditionally keeping to rigid allocation rules for the entire study (e.g. 1:1 allocation for treatment vs. control arms; Fig. 1C). Within adaptive randomization, there are three main types of randomization schemes: (a) *covariate-adaptive randomization*, where the goal is to balance the distribution of known covariates among the treatment arms; (b) *response-adaptive randomization*, where the goal is to increase the probability that patients will receive the treatment deemed to be most effective based on the cumulative data available at that instance; and (c) *covariate-adjusted response-adaptive randomization*, which utilizes features of both covariate- and response-adaptive randomization. Response-adaptive randomization schemes are appealing and are more ethical than traditional fixed allocation schemes because fewer patients are exposed to ineffective therapies [29]. Several implementations of response-adaptive randomization have been reported [50–52], including Bayesian adaptive procedures [49].

In 2012, an important simulation study by Trippa et al. retrospectively applied Bayesian response adaptive randomization to phase II clinical trials in recurrent GBM [53]. The authors found that if Bayesian adaptive randomization had been applied, 30 fewer patients could have been recruited while maintaining the same power level. Additionally, they simulated a multi-arm study design using Bayesian adaptive randomization with one control arm, two ineffective treatment arms with hazard ratios of 1.0, one effective treatment arm with a hazard ratio of 0.6, and a total sample size of 140 patients. For these conditions, the authors found that 12 more patients would be assigned to the effective treatment arm using the Bayesian adaptive randomization approach compared to conventional balanced designs (47 patients in adaptive treatment arm vs. 35 patients in balanced design treatment arm). The authors concluded that Bayesian adaptive randomization could be very valuable in GBM clinical trials and should be more widely adopted [53].

In 2020, Puduvalli et al. published the first GBM clinical trial to include Bayesian adaptive randomization and stopping rules [54••]. This multi-center phase II study assessed the efficacy of bevacizumab with or without vorinostat in patients with recurrent GBM. In the end, the trial was a negative trial that did not stop for efficacy with 41 patients assigned to the control arm and 49 patients assigned to the treatment arm. Nevertheless, this study was highly valuable to the field of neuro-oncology as it demonstrated the feasibility of Bayesian adaptive randomization in GBM clinical trials. The authors discussed the importance of developing a user-friendly and accessible program that can conduct the Bayesian adaptive design modifications; the need for constant collaboration between the physicians, biostatisticians, and the entire study team; and how the additional logistics involved in conducting an adaptive trial are far outweighed by the potential advantages in efficiency of adaptive designs over conventional study designs.

### Glioblastoma Adaptive Platform Trials: GBM-AGILE and INSIGHT

The GBM Adaptive Global Innovative Learning Environment (GBM AGILE; NCT03970447) [55] and the INdividualized Screening trial of Innovative GBM Therapy (INSIGhT; NCT02977780) [56] are two innovative and currently ongoing GBM clinical trials that extend adaptive designs to adaptive platform trial designs [57]. Briefly, adaptive platform trials [58, 59] can utilize response-adaptive randomization to simultaneously investigate multiple treatment arms, stop treatment arms based on success or futility, and add new experimental treatment arms during the course of the study (Fig. 1C). Adaptive platform trials can be highly advantageous in terms of efficiency because the same master protocol can be used to study multiple treatments (including combinations of treatments), multiple sources of financial support can be obtained given the numerous study drugs, and patient enrollment can be faster because only one control arm is needed for multiple treatments [58, 59].

Specifically, GBM AGILE can be considered a seamless phase II/III adaptive platform study for patients with newly diagnosed and recurrent GBM, and this trial is actively recruiting at the time of this manuscript’s preparation. Patients are allocated into treatment arms utilizing Bayesian adaptive randomization based on the overall survival outcomes of prior subjects, though progression-free survival may be later employed during the study [55]. Also, experimental therapies in GBM AGILE can “graduate” from phase II to a phase III study design upon demonstrated efficacy, and drug combination arms can be added too [55]. Although beyond the scope of this review, GBM AGILE also allows for biomarker-enrichment strategies (see review by Freidlin et al. [60]). A recent update showed that over 1000 patients have been screened for GBM AGILE so far, with enrollment rates 3–4 times greater than traditional GBM clinical trials in the past [61]. Furthermore, there are 46 active sites across the USA, Canada, and Switzerland. More sites are set to open in Germany, France, Switzerland, Italy, and Austria along with hopes to extend the trial to China and Australia [61].

The INSIGhT trial is a phase II adaptive platform trial that is very similar in design to GBM AGILE. The INSIGhT trial also includes Bayesian adaptive randomization and stopping rules for effective treatment arms. Some differences include that the study population is specifically newly diagnosed GBM with unmethylated O6-methylguanine–DNA methyltransferase (MGMT) gene promoter [56]. Also, there will be a maximum of 70 patients allocated to each treatment arm, Bayesian adaptive randomization will be performed utilizing progression-free survival [56], and there are 12 trial sites throughout the USA. Updates on the GBM AGILE and INSIGhT trials are eagerly awaited for both the results of treatment efficacies and the feasibility of adaptive platform trials in GBM.

### Statistical Considerations of Glioblastoma Adaptive Trials

It is important to consider Type I error probabilities and multiple comparisons corrections for innovative clinical trial designs [62], which can become complicated for adaptive designs because of interim analyses. In fact, the FDA final guidance on adaptive designs includes considerable discussion on the potential for inflated Type I error probabilities when performing the multiple hypothesis tests in clinical trials with adaptive designs (e.g., interim analyses, assessing multiple endpoints) as well as potential strategies to mitigate such errors [32]. In adaptive trials, prospectively-defining the interim analyses allows for adequately estimating type I error rates in adaptive trials [63], including for planning how ineffective therapies may be penalized in adaptive randomization schemes by not receiving as many patients while maintaining the same statistical rigor.

Additionally, the mathematical complexities of adaptive designs can be intimidating for non-biostatisticians. As a result, it is imperative that there remains close interaction and bridging between physicians and biostatisticians so that both fields can advance synergistically. For example, there can be increased development of user-friendly open-source software for adaptive designs—such as those from the MD Anderson Cancer Center Biostatistics Software Online website (https://biostatistics.mdanderson.org/softwareonline/)—so that clinical investigators can become familiarized with and utilize adaptive designs, which would consequently promote implementation of adaptive designs in future trials. To be clear, such educational materials and software do not replace the strong collaborations between physicians and biostatisticians that are required for clinical trials to be conducted, but rather they are a means to better facilitate the continued translation of novel statistical concepts into clinical trials through the highly vital collaborative efforts between physicians and biostatisticians.

## Conclusions

Phase 0, window of opportunity, and adaptive phase I–III studies offer unique advantages for GBM drug discovery. These novel clinical trial designs and their statistical considerations may allow for more efficient GBM clinical trials, allowing for less patients to receive ineffective therapies and the hope for more rapid discoveries of effective therapies for GBM.
